# Classification of Single Particles from Human Cell Extract Reveals Distinct Structures

**DOI:** 10.1016/j.celrep.2018.06.022

**Published:** 2018-07-03

**Authors:** Eric J. Verbeke, Anna L. Mallam, Kevin Drew, Edward M. Marcotte, David W. Taylor

**Affiliations:** 1 Department of Molecular Biosciences, University of Texas at Austin, Austin, TX 78712, USA; 2 Center for Systems and Synthetic Biology, University of Texas at Austin, Austin, TX 78712, USA; 3 Institute for Cellular and Molecular Biology, University of Texas at Austin, Austin, TX 78712, USA; 4 LIVESTRONG Cancer Institute, Dell Medical School, Austin, TX 78712, USA; 5 Lead Contact

## Abstract

Multi-protein complexes are necessary for nearly all cellular processes, and understanding their structure is required for elucidating their function. Current high-resolution strategies in structural biology are effective but lag behind other fields (e.g., genomics and proteomics) due to their reliance on purified samples rather than heterogeneous mixtures. Here, we present a method combining single-particle analysis by electron microscopy with protein identification by mass spectrometry to structurally characterize macromolecular complexes from human cell extract. We identify HSP60 through two-dimensional classification and obtain three-dimensional structures of native proteasomes directly from *ab initio* classification of a heterogeneous mixture of protein complexes. In addition, we reveal an ~1-MDa-size structure of unknown composition and reference our proteomics data to suggest possible identities. Our study shows the power of using a shotgun approach to electron microscopy (shotgun EM) when coupled with mass spectrometry as a tool to uncover the structures of macromolecular machines.

## In Brief

Verbeke et al. demonstrate a shotgun approach to macromolecular structure determination by combining single-particle electron microscopy with mass spectrometry to reconstruct multiple three-dimensional models in a single experiment. This approach provides a method for investigating the structure and function of cellular machinery in parallel.

## Graphical Abstract

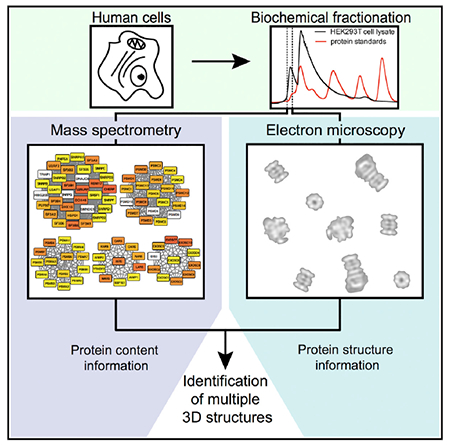

## INTRODUCTION

Protein complexes play an integral role in all cellular processes. Understanding the structural architecture of these complexes allows direct investigation of how proteins interact within macromolecular machines and perform their function. In an effort to understand which proteins assemble into these machines, proteome-wide studies have been conducted to determine the composition of protein complexes (Drew et al., 2017a; Gavin et al., 2002; Havugimana et al., 2012; Hein et al., 2015; Ho et al., 2002; Huttlin et al., 2015, 2017; Kastritis et al., 2017; Kristensen et al., 2012; Krogan et al., 2006; Wan et al., 2015). Similar studies have identified direct contacts between protein complex subunits computationally (Drew et al., 2017b) or by cross-linking mass spectrometry (Leitner et al., 2016; Liu and Heck, 2015; Rappsilber et al., 2000), and although these studies provide insightful predictions on protein-protein interactions, they lack directly observable structural information that can inform us on function and subunit stoichiometry.

Structural genomics approaches, such as the Protein Structure Initiative, have thus far been the most successful way to systematically solve structures for proteins lacking a model (Chandonia and Brenner, 2006). These approaches have removed several bottleneck steps in traditional structural biology by applying high-throughput technology to sample preparation, data collection, and structure determination. Although many high-resolution structures have resulted from structural genomics, these approaches typically miss large complexes and perform best on single proteins or low-molecular-weight complexes that can be purified and crystallized for X-ray crystallography or labeled for nuclear magnetic resonance (Montelione, 2012).

Recent advances in electron microscopy (EM) software and hardware have dramatically increased our ability to solve the structures of native protein complexes and allow for increased throughput approaches using EM. Automated microscopy software, such as Leginon (Suloway et al., 2005), SerialEM (Mastronarde, 2005), and EPU (FEI), allow for the collection of large datasets in a high-throughput, semi-supervised manner. RELION, a Bayesian algorithm for 3D classification, allows users to sort conformationally heterogeneous samples to define structurally homogeneous classes (Scheres, 2012). Furthermore, 3D reconstructions can now be done *ab initio* (without an initial model) by a computationally unsupervised approach using cryoSPARC (Punjani et al., 2017). These strategies potentially allow for analysis of heterogeneous mixtures, although this aspect has not been explored extensively.

Advances in hardware, such as direct electron detectors and Volta phase plates, allow visualization of particles at near atomic resolutions and smaller molecular weights, which was previously only possible for larger particles or particles with high symmetry (Danev and Baumeister, 2016; Kühlbrandt, 2014). Despite these revolutionary advances, single-particle EM is still largely used to study homogeneous samples, where the identity of the protein complex is known *a priori*.

Here, we take a different approach to structure determination by exploiting advances in EM software to structurally classify native protein complexes from human cell lysate. By using a shotgun approach to EM (shotgun EM), we chromatographically separate cell lysate into tractable fractions before identification by mass spectrometry (MS) and structural analysis by EM. Using this approach, we characterize compositionally and structurally heterogeneous protein complexes from immortalized (HEK293T) cells separated by macromolecular size using size-exclusion chromatography (SEC).

For this study, we determined the protein composition of two different high-molecular-weight samples from SEC by MS experiments. Identified proteins were then mapped to previously generated protein interaction networks to reveal candidate protein complexes. We then collected negative-stain EM data and performed single-particle analysis of heterogeneous particles simultaneously. Using this approach, we identified structurally distinctive macromolecular machines after unbiased 3D classification and *ab initio* reconstruction of single particles.

## RESULTS

### Separation and Identification of Subunits from High-Molecular-Weight Protein Complexes

**Figure 1.**
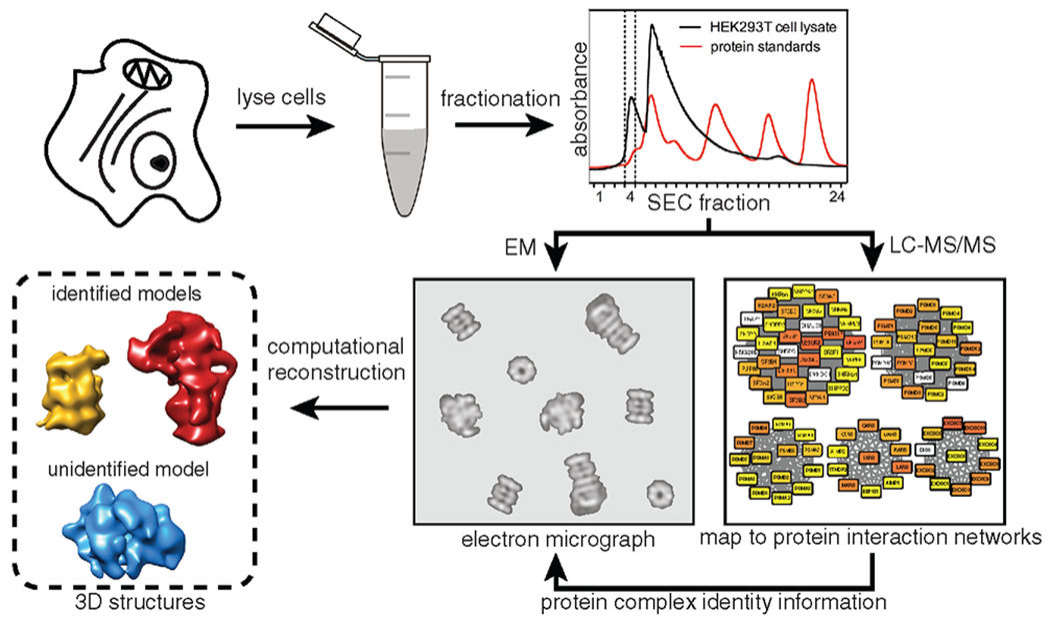
Shotgun EM Pipeline Used for Structural Determination of Multiple Macromolecular Complexes HEK293T cells are subjected to lysis and separation using SEC. The resulting fractions are characterized separately by electron microscopy and mass spectrometry. Proteins identified from mass spectrometry are mapped to known and predicted protein complexes to identify which complexes are present in a given fraction. Electron microscopy data are then used to generate structures of multiple protein complexes.

**Figure 2.**
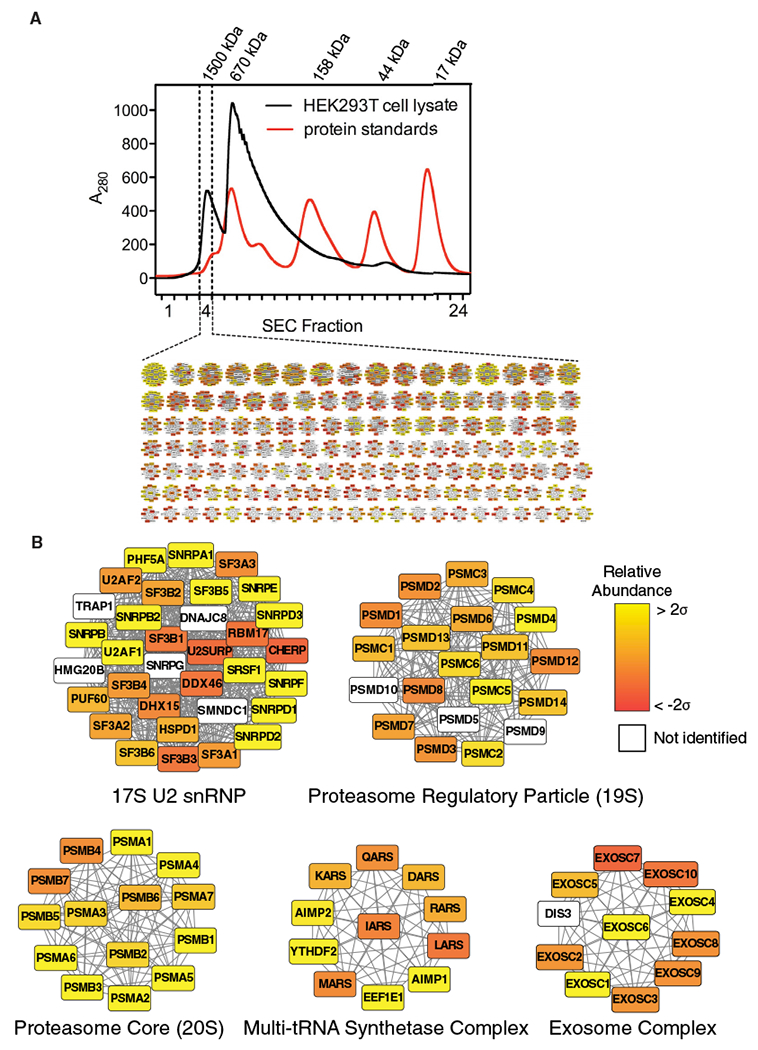
Identification of Protein Complexes in a Cellular Fraction (A) Elution profile from SEC. Elution profiles of protein standards are overlaid to estimate the molecular weight range of protein complexes in fraction 4. Inset: a network map displaying a portion of the 1,375 candidate complexes determined by mapping mass spectrometry data to combined protein interaction networks is shown. (B) Enlarged view of a subset of candidate complexes. A filled node indicates a protein was identified by mass spectrometry; a white node indicates the protein was not identified. Color gradation of filled nodes indicates the relative abundance (determined by label-free quantification) ranging from ±2 SDs. See also Figure S1 and Table S1.

Native macromolecular assemblies from lysed human cells were first separated by macromolecular size using SEC (see STAR Methods). We selected a high-molecular-weight fraction (fraction 4) for MS and EM analysis (Figure 1) with molecular weights in the range of 1.5 to 2 MDa based on molecular standards (Figure 2A; see STAR Methods).

MS analysis of our sample (Figure 2A) identified 1,401 unique proteins. Over 93% of the identified proteins had a molecular weight under 200 kDa, indicating that the proteins are likely multi-subunit complexes in order to elute in the high-molecular-weight fraction. We then mapped the proteins identified by MS to a combined set of protein-protein interaction networks to suggest the identity of complexes in our sample (Figure 2B). The previously determined protein-protein interaction networks include hu.MAP (Drew et al., 2017a) and CORUM (Ruepp et al., 2010), which were chosen to provide a list of documented and high-confidence protein complexes. Furthermore, hu.MAP incorporates datasets from previous interactome studies (Havugimana et al., 2012; Hein et al., 2015; Huttlin et al., 2015; Wan et al., 2015) and includes greater than 4,000 complexes. In addition, we incorporated interaction networks that exclusively used size-exclusion chromatography and quantitative proteomics to determine protein-protein interactions (Kristensen et al., 2012; Larance et al., 2016). The combined protein interaction network included 7,021 protein complexes. We identified specific, well-annotated protein complexes within our sample, which contains both structurally defined complexes (e.g., the proteasome; Lander et al., 2012; Schweitzer et al., 2016) and complexes without known structures (e.g., the multi-tRNA synthetase complex; Mirande, 2017; Figure 2B).

Complexes with at least 50% of their subunits identified were kept as candidates for subsequent analysis. Many of the resulting candidate complexes shared a number of individual subunits and are different variants of the same complex. In order to group related complexes, we created a hierarchical network by performing an all-by-all comparison of proteins between each complex (Figure S1; see STAR Methods). Our hierarchies suggest we have 234 groups of related complexes (i.e., with shared subunits) in addition to the remaining 538 unique complexes for a total of 772 complexes in our sample (Table S1).

The abundance of each complex was then calculated using two different label-free quantification strategies to rank the predicted complexes that might be visible by EM. Both normalized spectral counting (Vaudel et al., 2015) and top 3 extracted ion chromatogram areas (Silva et al., 2006; see STAR Methods) produced similar abundance values for each protein complex (Figure S1). By combining our hierarchical network with the relative abundance for each complex, we identified the specific subunit composition of complexes most likely to be present in our sample. As an example, we can examine the group of related proteasome complexes (Figure S1), showing many related complexes, where the canonical 26S proteasome appears to be the most abundant form. This analysis reveals complexes of interest in our sample, which vary in abundance.

### EM of Single Particles from HEK293T Cell Extract Fraction

**Figure 3.**
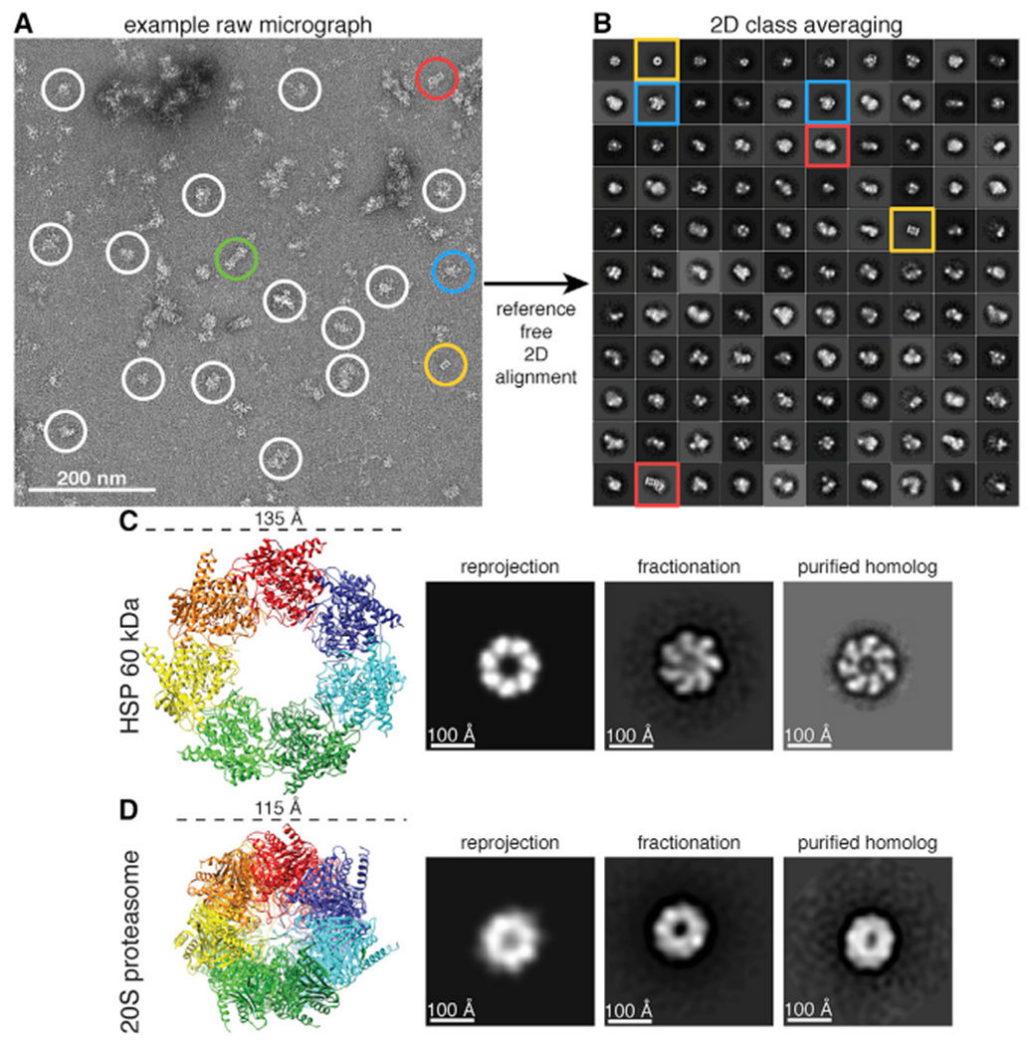
Structural Characterization of Protein Complexes from Cell Extract (A) Raw micrograph of negatively stained sample from SEC. Proteasome particles in three different biochemical forms, 20S core, single-capped 26S (20S core with one 19S regulatory particle), and double-capped 26S (20S core with two 19S regulatory particle), are circled in gold, red, and green, respectively. Representative unidentified particles are circled in white. Class averages with well-resolved structural features are circled in blue. (B) Reference-free 2D class averages of 31,731 template-picked particles generated using RELION. The size of each box is 576 × 576 Å. The 2D class averages are sorted in decreasing order based on the number of particles belonging to a class, with 110 out of 300 2D classes shown. (C) Crystal structure of HSP60 (PDB: 4PJ1) identified by MS and its corresponding reprojection after being low-pass filtered to 30 Å. The 2D class average from our fractionation (fraction 8) matching both the reprojection and a class average of a negatively stained purified homolog (GroEL), adapted from Danziger et al. (2003), suggests the identity of our 2D class average as HSP60. Image box sizes are scaled for consistency. (D) Crystal structure of the 20S proteasome (PDB: 4R30) and its corresponding reprojection after being low-pass filtered to 30 Å. The 2D class average from our fractionation (fraction 4) matching both the reprojection and a class average of a negatively stained, purified *S. cerevisiae* proteasome suggests the identity of our 2D class average as the 20S proteasome. Image box sizes are scaled for consistency. See also Figure S2.

Having identified candidate complexes in our sample by MS, we next use negative-stain EM to investigate the structures of the complexes. Negative-stain EM samples are easily prepared and are often used to determine the heterogeneity of a sample because of the higher signal-to-noise ratio compared to cryo-EM. Raw micrographs of our negatively stained sample show monodisperse particles with clear structural features (Figure 3A). Intact, structurally heterogeneous complexes can be directly observed. The proteasome can be seen in three different structural states, as a core (20S), as a single-capped proteasome (20S core with one 19S regulatory particle), and as a double-capped proteasome (26S, 20S core with two 19S regulatory particles). In addition, many other unidentified particles can be clearly seen, with an average particle diameter of ~200 Å.

Template picking from 1,250 micrographs of our sample resulted in a final set of 31,731 particles after filtering out ~67% of particles as “junk” particles (see STAR Methods). To assess the quality of automated template picking, we also manually selected 35,381 particles for alignment and classification. A comparison of the reference-free 2D class averages of both manually and template-picked datasets yielded similar results (Figure S2), and both datasets were used for independent downstream processing. 2D class averages yielded distinct class averages with various morphologies and features. Remarkably, many well-defined classes emerged from this heterogeneous mixture of complexes (Figure 3B).

Interestingly, we observed two distinct heptameric rings in our reference-free 2D classification (Figures 3C and 3D). One of the rings is wider in diameter with a pinwheel-like architecture (Figure 3C), and the second is rounder and narrower (Figure 3D). To uncover the identity of these rings, we turned to our mass spectrometry data for candidate ring-forming complexes. Two of the identified complexes, heat shock protein 60 (HSP60) and the α and β rings of the proteasome core, are known to form heptameric rings. The X-ray crystal structures of both HSP60 and the proteasome core were used to compare to our candidate structures. HSP60 is 135 Å in diameter (PDB: 4PJ1; Nisemblat et al., 2015), and the ring of the 20S core (PDB: 4R3O; Harshbarger et al., 2015) is 115 Å in diameter, which suggested an identity for each of the rings by a comparison of diameters. To test this hypothesis, we reprojected the X-ray crystal structure of both protein complexes after low-pass filtering to 30-Å resolution to simulate 2D projections and compared them to our class averages. Finally, we compared reference-free class averages of purified GroEL (Danziger et al., 2003; a well-studied HSP60 homolog) and proteasome core to our fractionation data. All of these comparisons provide strong evidence that the pinwheel-like and narrow ring projections correspond to HSP60 and the proteasome core, respectively.

To further validate our identification of HSP60, we performed negative-stain EM on a second fraction from our SEC, fraction 8, where HSP60 was also identified by mass spectrometry. The approximate molecular weight of native macromolecular assemblies in fraction 8 is 500 kDa (Figure 2A). For particle selection of fraction 8 EM data, we used a difference-of-Gaussian picker (Voss et al., 2009). This method was chosen as an orthogonal, reference-free method to independently confirm whether we could identify HSP60. Reference-free 2D class averages obtained using this particle-picking scheme revealed a class average with a well-defined pinwheel-like architecture (Figure S2), suggesting HSP60 was also identified in fraction 8.

### 3D Classification of a Heterogeneous Mixture Produces Distinct Structures

**Figure 4.**
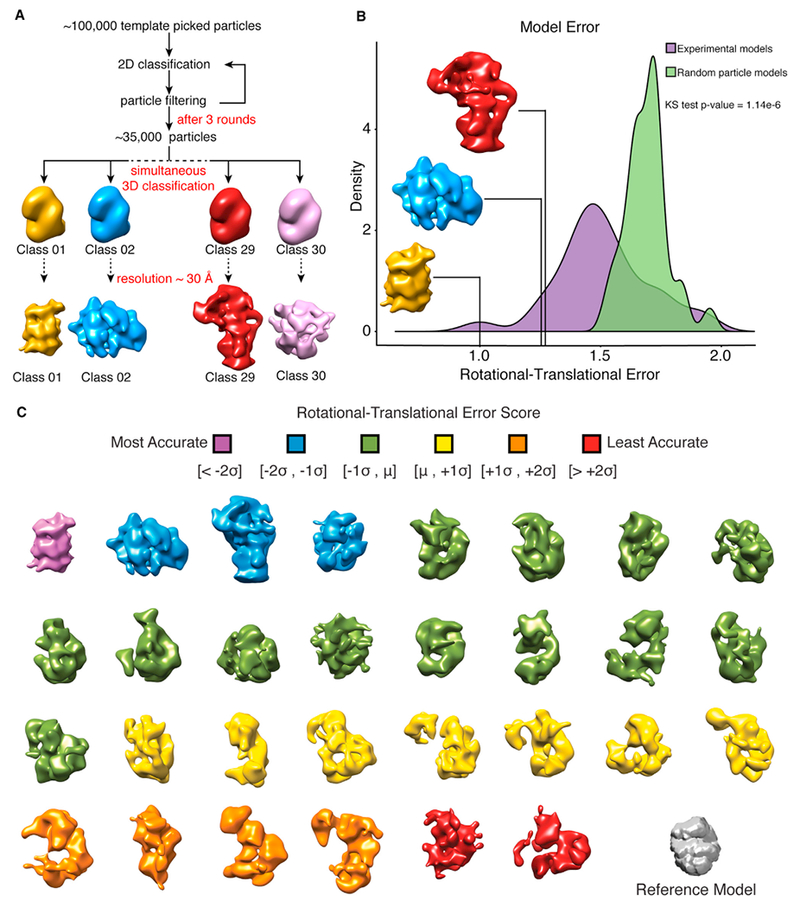
Classification of Distinct Protein Complex Architectures (A) Classification workflow for the simultaneous generation of 30 3D models from the complete dataset of particles using RELION. Models were built using DNA-dependent protein kinase catalytic subunit low-pass filtered to 60 Å as an arbitrary reference model. (B) Top 3 models generated using RELION. Models were scored based on their rotational-translational error (a measure of the internal consistency of the model; see STAR Methods). The distribution of model error scores was compared to models generated using random particles from our template-picked data. (C) 30 classes generated using RELION from the complete template-picked dataset of particles with the reference model shown in gray. Models are colored by their rotational-translational error and are unrelated to colors in (A) and (B). See also Figure S3.

Given the success of 2D classification at separating particles into distinct classes, we then performed 3D classification on the entire set of particles using RELION (Scheres, 2012) to simultaneously generate 30 reconstructions (Figure 4A). Whereas RELION was developed to group 2D projections of the same protein or protein complex with conformational heterogeneity into distinct classes, we asked whether RELION could also classify projections from many distinct complexes in a heterogeneous mixture into internally consistent (low-error) reconstructions.

To test the internal consistency of the 3D reconstructions, we determined the distribution of calculated error within the models and ranked each reconstruction based on a rotational-translational error score (see STAR Methods). The error score distribution was then compared to the rotational-translational error scores of models built from random particles in the dataset to evaluate our ability to classify related particles belonging to a particular model and demonstrated our 3D reconstructions have substantially less error than random reconstructions (Figure 4B). The 30 3D reconstructions generated all contained various degrees of structural details ranging from distinct barrels to more globular shapes (Figure 4C), suggesting it is possible to classify particles from a heterogeneous mixture into distinct structures.

We then performed cross-correlations between our top 3 models and several complexes with known structure from our MS-determined list of high-abundance complexes to determine whether we could link our structural models with complex identity (Figure S3; see STAR Methods). The 20S proteasome emerges as a clear match when compared to our highest scoring model with a cross-correlation score of 0.87. We were also able to distinguish a single-capped proteasome, which matched to our third highest scoring model with a cross-correlation score of 0.81. Interestingly, our second highest scoring model was not readily recognizable, and none of the known structures emerged as a clear match after cross-correlation. Based on the high-abundance 2D class averages and large volume of the unknown complex, we filtered our proteomics data to search for possible identities. Our search suggests the unknown complex is likely a variant of a mitochondrial ribosome, spliceosome, or DNA-repair complex, but given the current resolution, the results are inconclusive. A much larger set of particles or projections and deeper classification is likely required for assignment of this structure. However, our results suggest it is possible to solve multiple structures from cell lysate in a parallel manner, even in the absence of matching starting models.

### Quantification and *Ab Initio* Reconstruction of the Proteasome

To determine our ability to further characterize complexes identified in a complex mixture, we investigated our sample specifically in the context of the proteasome, which allowed us to evaluate the success of reconstructions without an initial model. Our goals were to (1) investigate whether *ab initio* reconstructions would reveal clear proteasome structures, (2) determine the ratio of the 20S core and single-capped proteasomes using our single-particle data, and (3) compare single-particle counting of the proteasome to label-free MS quantification.

**Figure 5.**
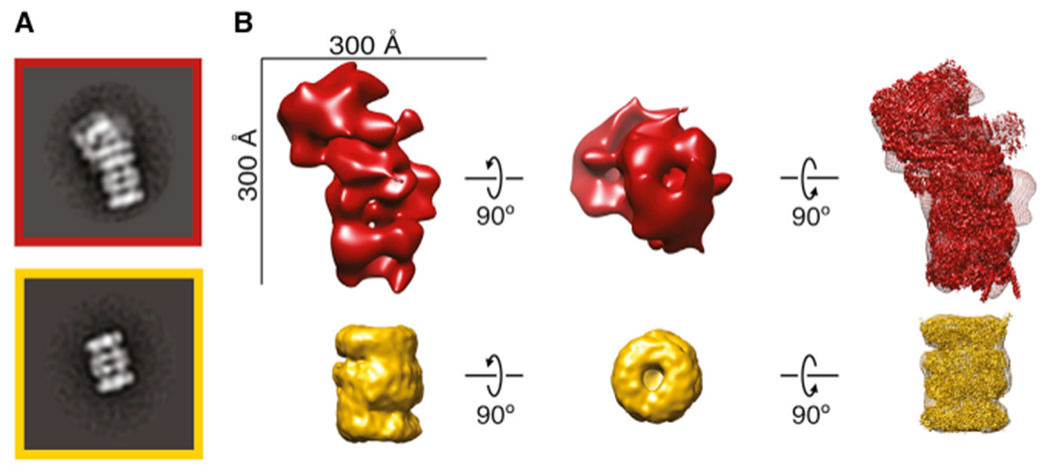
*Ab Initio* Structures from a Cellular Fraction Unambiguously Reveal the Proteasome (A) Reference-free 2D class averages of the proteasome from Figure 3B. (B) Top: structure of single-capped proteasome generated using RELION from manually picked particles. Bottom: *ab initio* structure of the 20S core proteasome generated using cryoSPARC is shown. High-resolution structures EMD-4002 (Schweitzer et al., 2016) and EMD-2981 (da Fonseca and Morris, 2015) are fit into the structures, respectively. See also Figures S4 and S5.

Class averages of the 20S core and single-capped proteasomes were clearly identified as barrel-shaped particles and barrels with large rectangular caps, respectively (Figure 5A). Based on identifying the proteasome with notably distinct 2D class averages, as well as RELION-based 3D classification producing two identifiable proteasome models, we asked whether *ab initio* reconstructions were capable of correctly recovering proteasome structures. We therefore attempted a completely unsupervised approach for 3D classification using cryoSPARC (Punjani et al., 2017). cryoSPARC was developed for determining multiple 3D structures of a protein without prior structural knowledge or the assumption that the ensemble of conformations resembled each other, but in this context, we evaluated its ability to classify 2D particles of distinct complexes in a mixture. Remarkably, a 3D reconstruction of the 20S core was generated using *ab initio* reconstruction in cryoSPARC on the entire dataset of particles with 5, 10, and 15 classes (Figure S4).

From the structures generated with 10 classes, a distinct 3D reconstruction of the 20S core showing a clear barrel with a central channel and some separation of co-axial rings was produced (Figure 5B). This 20S core reconstruction contains 3,150 particles with an estimated resolution of 20.4 Å using the 0.143 Fourier shell correlation (FSC) criterion (Figure S4). Our 3D map is consistent with a recent high-resolution structure of the 20S core (EMD-2981; da Fonseca and Morris, 2015) with a cross-correlation score of 0.94.

We were unable to distinguish a 3D structure of the single-capped proteasome from cryoSPARC. However, going back to our single-capped proteasome from 3D classification using RELION, we were able to dock in a high-resolution structure determined previously (EMD-4002; Schweitzer et al., 2016; Figure 5B). The high-resolution structure can be unambiguously docked into our EM density (cross-correlation score of 0.76) albeit with less agreement given the low number of particles in the model (1,121 particles). Using RELION to refine the structure of our single-capped proteasome, we achieved a nominal resolution of 31 Å (Figure S4).

We then quantified the ratio of 20S core to single-capped proteasome particles by directly counting individual particles from our EM data of fractionated cell lysate. Revisiting our 2D classification, we compared the number of particles aligned in the side view of the 20S core and single-capped proteasome (Figure 5A). The ratio of 20S core to single-capped proteasome particles in our sample was calculated to be 3:2 or 1 bound 19S regulatory particles for every 2.5 20S core particles in our sample by EM. This is similar to our MS data, which suggest the ratio of 19S regulatory particles to 20S core particles is 1:1 (Figure S5). Collectively, our study suggests it is not only possible to solve structures of protein complexes from cell lysate *ab initio* but also quantify the stoichiometry of biochemical states.

## DISCUSSION

One bottleneck of structural biology is the current limitation of studying only a single protein or protein complex structure in a single experiment. However, recent advances in detectors and software for EM bring about the possibility of high-throughput structural determination using EM. To this end, we have demonstrated shotgun EM as a potential pipeline for high-throughput identification and structural determination of macromolecular machines. By combining MS and EM, we demonstrate it is possible to structurally characterize and identify protein complexes from a cellular sample containing many native complexes. This pipeline was used to successfully identify the proteasome in two biochemical forms and HSP60 from a cellular fraction with minimal user input. HSP60 was then independently verified through another SEC fraction identified as containing HSP60 by MS. Additionally, we construct a self-consistent structural model of an ~1-MDa protein complex of unknown identity.

A recent study showed that higher order assemblies from a eukaryotic thermophile could be separated chromatographically, identified by MS, and visualized through cryo-EM to obtain a high-resolution structure (Kastritis et al., 2017). The authors performed cryo-EM on particles from a complex mixture to solve a 4.7-Å-resolution structure of fatty acid synthase from cell lysate separated by molecular size after a 50% enrichment for fatty acid synthase. In our study using human cells, which have a canonical proteome approximately 3 times larger than *C. thermophilum,* we are able to obtain structural information from a complex mixture without enrichment, suggesting that sample heterogeneity is a surmountable problem. A combined approach using shotgun EM and the cryo-EM protocol presented by Kastritis et al. (2017) provides a potential strategy for recovering multiple high-resolution structures from fractionated cellular extracts.

Several key barriers to structurally classifying heterogeneous mixtures remain, with the main challenge being to correctly assign different orientations of the same complex in large datasets of heterogeneous mixtures. Additionally, assigning the correct subunit composition to the unidentified molecular models (UMMs) uncovered using shotgun EM, particularly for complexes lacking structural information, will present a unique challenge to structural biology. Whereas currently we cannot identify each class average or 3D structure obtained in this study, we are able to distinguish different structural states of the proteasome using current *ab initio* methods, suggesting that shotgun EM is a promising tool to characterize the heterogeneity of protein complex forms. Our top-scoring UMM was not readily recognizable and had no apparent match from model fitting. It is possible our model has been structurally annotated previously but was not covered in our search. Alternatively, it is possible our model remains unidentified because it is structurally novel. In future experiments, a comprehensive list of solved structures coupled with optimal volume alignment and cross-correlation can be used to identify likely matches to models generated using shotgun EM.

One challenge when dealing with protein complexes is defining their precise subunits. MS does not indicate which complex a protein belonging to multiple complexes was identified from. Many of these related complexes and sub-complexes have yet to be structurally or biochemically characterized. Our hierarchical network strategy allows us to make an initial estimate on which form of a complex might be in our EM data. Using shotgun EM, we aim to validate these uncharacterized and other less-characterized forms of complexes that may be more amenable to our separation scheme.

A key proof of concept in this study was the proteasome, which is a structurally distinct complex and serves a crucial role in protein degradation in eukaryotic cells (Finley, 2009). The native stoichiometry of the proteasome has been studied in different ways by multiple groups (Asano et al., 2015; Havugimana et al., 2012). Our template-picked counting of single proteasome particles has an advantage over MS approaches by identifying which form of a complex an identified protein belongs to. Although our MS and EM quantification were similar, showing an approximate ratio of 20S core to 19S regulatory particles ranging from 1:1 to 2:1, a separate study using corrected spectral counts suggests the ratio is closer to 4:1 (Havugimana et al., 2012). To reconcile these two observations, more chromatographic fractions containing the proteasome would need to be quantified by EM and MS to see whether there is agreement. As more protein complexes become structurally annotated, shotgun EM can be used as an auxiliary method for quantifying the abundances of native complexes, as well as their stoichiometry.

After *ab initio* 3D classification, we obtained a reasonable reconstruction of the 20S core in cryoSPARC from 3,150 particles. Although only half of these particles are accounted for from 2D class averaging of all particles, it is likely that the discrepancy results from proteasome particles that are misclassified or exist in different, less-populated orientations in our 2D class averages. Alternatively, because the number of models we could reconstruct in 3D was limited by the small populations of each complex we had in our micrographs, it is possible that non-proteasome particles were grouped into our 3D class of the proteasome. These misclassified particles would have a small contribution to the overall likelihood of the 3D map as it is reconstructed (Punjani et al., 2017). One method to separate misclassified particles would be to do iterative rounds of 3D classification.

In this study, we used a 60S ribosome class average as a template for auto-picking due to its large molecular weight and round shape. Interestingly, none of the resulting averages resembled the 60S, providing evidence that we were not biasing the results from template picking and subsequent data analysis. A similar concern for model bias exists when using RELION to generate 3D models. Despite this, none of the 3D classes are visually identical to the reference 3D model, with most EMD structures selected from our MS data outscoring the reference model by cross-correlation score when compared to our top 3 RELION models. In future experiments, more sophisticated template matching, deep learning algorithms, or *ab initio* methods can be introduced to improve particle identification and model building (Punjani et al., 2017; Rickgauer et al., 2017; Wang et al., 2016).

This study represents an advance into structural proteomics using EM, suggesting that parallel structural determination of protein complexes shows promise for alleviating bottlenecks in structural biology. In the interim before high-resolution data are collected, it is possible to search for structurally uncharacterized complexes through the addition of protein tags (Flemming et al., 2010) to identify complexes in a heterogeneous mix without the need to purify the sample. One could also utilize integrative structural biology approaches to have a predicted model with which to search for structures in cell extract. We envision using cryo-EM for this pipeline to solve sub-nanometer-resolution structures, where homology models and known structures can be more clearly compared. Moving this pipeline to cryo-EM will likely aid in our identification of candidate complexes; however, several obstacles will need to be overcome, including (1) lower signal-to-noise ratio, (2) complex instability (i.e., protein complexes being degraded into non-native compositions), and (3) the increased amount of data required for reconstructions. Future studies will be required to determine whether we can overcome these potential pitfalls when transitioning the pipeline into cryo-EM.

Shotgun EM will accelerate the pace at which structural information is generated and allow us to better understand the structure-function relationship of proteins. Optimization of this technique has the potential to address questions about many macromolecular machines across different cell types, disease states, and species. We propose that investigating the collective protein complexes in a cell, or the “complexome,” using shotgun cryo-EM will help inform us broadly on systems biology, cell biology, and changes in complexes that contribute to human diseases.

## STAR★METHODS

### KEY RESOURCES TABLE

**Table:** 

REAGENT or RESOURCE	SOURCE	IDENTIFIER
Deposited Data		
20S Proteasome Core	This paper	EMDB: EMD-7946
26S Single-capped Proteasome	This paper	EMDB: EMD-7947
Fraction 4 proteomics data	This paper	PRIDE: PXD010026
Experimental Models: Cell Lines		
HEK293T	ATCC	CRL3216
Software and Algorithms		
Appion	(Lander et al., 2009)	http://nramm.nysbc.org/software/
FindEM	(Roseman, 2004)	N/A
DoG Picker	(Voss et al., 2009)	N/A
Proteome Discoverer	ThermoFisher Scientific	https://www.thermofisher.com/order/catalog/product/OPTON-30795
RELION	(Scheres, 2012)	http://www2.mrc-lmb.cam.ac.uk/relion/index.php?title=Main_Page
CTFFIND4	(Rohou and Grigorieff, 2015)	http://grigoriefflab.janelia.org/ctf
cryoSPARC	(Punjani et al., 2017)	https://cryosparc.com/
Chimera	(Pettersen et al., 2004)	https://www.cgl.ucsf.edu/chimera/
SearchGUI	(Vaudel et al., 2011)	http://compomics.github.io/projects/searchgui.html
PeptideShaker	(Vaudel et al., 2015)	http://compomics.github.io/projects/peptide-shaker.html
Cytoscape	(Shannon et al., 2003)	http://www.cytoscape.org/
Other		
Formvar/Carbon 400 mesh, Copper approx. grid hole size: 42μm	Ted Pella	01754-F

### CONTACT FOR REAGENT AND RESOURCE SHARING

Further information and requests for resources and reagents should be directed to and will be fulfilled by the Lead Contact, David W. Taylor dtaylor@utexas.edu (D.W.T.).

### EXPERIMENTAL MODEL AND SUBJECT DETAILS

HEK293T cells (ATCC CRL3216) cultured at 37°C in DMEM (GIBCO) supplemented with 10% (v/v) FBS (Life Technologies) were continually split over 7 days to give four 10-cm dishes of adherent cells.

### METHOD DETAILS

#### Cell Culture and Extract Preparation

HEK293T cells were harvested at 80%–100% confluence without trypsin by washing in ice cold phosphate buffered saline (PBS) pH 7.2 (0.75 mL; GIBCO) and placed on ice. Cells (approximately 10 mg) were lysed on ice (5 min) by resuspension in Pierce IP Lysis Buffer (0.8 mL; 25 mM Tris-HCl pH 7.4, 150 mM NaCl, 1 mM EDTA, 1% NP-40 and 5% glycerol; Thermo Fisher) containing 1× protease inhibitor cocktail III (Calbiochem). The resulting lysate was clarified (17,000 g, 10 min, 4°C) and filtered (Ultrafree-MC filter unit (Millipore); 12,000 g, 2 min, 4°C).

#### Biochemical Fractionation Using Native Size-Exclusion Chromatography

Size-exclusion chromatography (SEC) was performed at 4°C on an AKTA FPLC (GE Healthcare). Approximately 6 mg of soluble protein was applied to a Superdex 200 10/300 GL analytical gel filtration column (GE Healthcare) equilibrated in PBS, pH 7.2 at a flow rate of 0.5 mL min^−1^. Fractions were collected every 0.5 mL. The elution volumes of molecular weight standards (Thyroglobulin, 670,000 Da; γ-globulin, 158,000 Da; Ovalbumin, 44,000 Da; Myoglobin, 17,000 Da; Vitamin B_12_, 1,350 Da; Biorad) were additionally measured to calibrate the column (Figure 2A). Fraction 4 (concentration ~1 mg/mL) was deemed most likely to contain a high number of large complexes, as determined by A_280_, and was subjected to further proteomic and structural analysis.

#### Mass Spectrometry

50 μL of Fraction 4 (Figure 2A) was denatured and reduced in 50% 2,2,2-trifluoroethanol (TFE) and 5 mM tris(2-carboxyethyl)phosphine (TCEP) at 55°C for 45 minutes, followed by alkylation in the dark with iodoacetamide (55 mM, 30 min, RT). Samples were diluted to 5% TFE in 50 mM Tris-HCl, pH 8.0, 2 mM CaCl_2_, and digested with trypsin (1:50; proteomic grade; 5 hours: 37°C). Digestion was quenched (1% formic acid), and the sample volume reduced to ~100 μL by speed vacuum centrifugation. The sample was washed on a HyperSep C18 SpinTip (Thermo Fisher), eluted, reduced to near dryness by speed vacuum centrifugation, and resuspended in 5% acetonitrile/ 0.1% formic acid for analysis by liquid chromatography tandem mass spectrometry (LC-MS/MS). Peptides were separated on a 75 μM × 25 cm Acclaim PepMap100 C-18 column (Thermo) using a 3%–45% acetonitrile gradient over 60 min and analyzed on line by nanoelectrospray-ionization tandem mass spectrometry on an Orbitrap Fusion (Thermo Scientific). Data-dependent acquisition was activated, with parent ion (MS1) scans collected at high-resolution (120,000). Ions with charge 1 were selected for collision-induced dissociation fragmentation spectrum acquisition (MS2) in the ion trap, using a Top Speed acquisition time of 3 s. Dynamic exclusion was activated, with a 60 s exclusion time for ions selected more than once.

#### Proteomic and Bioinformatics Analyses

The mass spectrometry data were processed independently using searchGUI and PeptideShaker (Vaudel et al., 2011,^2015^) and Proteome Discoverer (ThermoFisher Scientific). Data were searched against a target-decoy human database downloaded from Universal Protein Resources Database (UniProtKB/Swiss-Prot comprising human proteins supplemented with common contaminants). Fixed modifications of carboxyamidomethylated cysteine and variable modifications of oxidized methionine and acetylation of protein N terminus were permitted to allow for detection of modified peptides. Peptide spectral matches, peptides and proteins were considered positively identified if detected within a 1% false discovery rate cut off (based on empirical target-decoy database search results). Additionally, proteins were only considered for further processing if at least one unique peptide was identified. This screening procedure resulted in 1,402 distinct human proteins. To facilitate mapping to a protein ID, we used UniProtKB accession numbers as a common identifier and the UniProt ID mapping tool to interconvert different gene and protein identifiers.

Relative abundance for each complex was determined using two different methods of label-free quantification, one calculated using peptide spectral matches and the other calculated using extracted ion chromatogram area (XIC). Protein length was used for normalizing the number of peptide spectral matches observed for each protein using the Normalized Spectral Abundance Factor (NSAF) as calculated by PeptideShaker (Vaudel et al., 2015). Proteins expected to participate in a complex as predicted by our combined protein interaction network, which were not identified by MS, were assigned a NSAF value of zero. The NSAF values for all proteins in a complex were then averaged to estimate the relative abundance of each complex.

To calculate relative abundance based on XIC, each protein was assigned an abundance by taking the average of the top-3 peptide areas identified for that protein using Proteome Discoverer (ThermoFisher Scientific). Proteins expected to participate in a complex as predicted by our combined protein interaction network, which were not identified by MS, were assigned an abundance of zero. The average area values for all proteins in a complex were then averaged to estimate the relative abundance of each complex.

The hierarchical network of protein complexes in Figure S1 was created by determining the percent of shared subunits between all complexes. For a predicted protein complex A with subunits {*a*_1_, *a*_2_, …, *a_n_*} and B with subunits {*b*_1_, *b*_2_, …, *b_m_*}, the similarity score (S) of A to B was calculated by finding the intersection of A and B divided by the size of set A as follows (Equation 1).

(1) $$S=\frac{\left|A\cap B\right|}{\left|A\right|}$$

If the similarity score between complexes was 90% or greater, it was considered a related complex. The resulting network shows related groups of complexes where at least 90% of subunits in higher-order complexes are shared between sub-complexes. 837 of the 1375 complexes identified by MS belong to a group of shared complexes. Furthermore, the 837 shared complexes in our sample can be organized into 234 distinct hierarchies. The network of related complexes was then visualized using Cytoscape with edges corresponding to the similarity score (Shannon et al., 2003).

#### Negative Stain Electron Microscopy Sample Preparation

4 μL of fractionated human cell lysate was applied to a glow-discharged 400-mesh continuous carbon grid. After a 1 min adsorption, the sample was negatively stained with five consecutive droplets of 2% (w/v) uranyl acetate solution, blotted to remove residual stain, and air-dried in a fume hood.

#### Electron Microscopy

Data was acquired using a JEOL 2010F transmission electron microscope operated at 200 keV with a nominal magnification of ×60,000 (3.6 Å at the specimen level). Each image was acquired using a 1 s exposure time with a total dose of ~30–35 e^−^ Å^−2^ and a defocus between −1 and −2 μm. A total of 1,250 micrographs were manually recorded on a Gatan OneView.

#### 3D Reconstruction and Analysis

Two independent particle stacks were generated from the same 1,250 micrographs using either template or manual particle picking. The contrast transfer function (CTF) of each micrograph was estimated using CTFFIND4 (Rohou and Grigorieff, 2015). FindEM (Roseman, 2004) was used for template-based particle picking using a reference-free 2D class average of our negatively stained 60S Ribosome from *Saccharomyces cerevisiae* (a gift from A. Johnson). We chose this template for particle picking as it picked virtually all particles in each micrograph. It would also be easily recognizable in class averages if there were a template bias. Importantly, none of the resulting class averages matched this ribosome. ~97,000 and ~37,000 particles were selected by template picking and manually selecting particle images, respectively. All image pre-processing was done in Appion (Lander et al., 2009). After removing junk particles, 31,731 particles were left from template picking and 35,381 particles from manual picking, respectively. The majority of junk classes from template picking can be attributed to the picking of particles within aggregates and two particles as one. Particle box size was set to 576 Å × 576 Å. For our second fraction analyzed by EM (fraction 8), particles were selected in an automated manner using a Difference of Gaussian (DoG) particle picker (Voss et al., 2009). ~75,000 particles were picked from 300 micrographs. Junk particles were filtered from the dataset resulting in a final set of 28,553 particles. Particle box size was set to 518.4 Å × 518.4 Å.

Reference-free 2D class averages were generated with 300 classes for both fraction 4 and fraction 8 datasets using RELION (Scheres, 2012). Next, 3D classification was performed on fraction 4 data using RELION to create 30 classes of both datasets. The structure of DNA-dependent protein kinase catalytic subunit was chosen as an initial model using a negative stain structure low-pass filtered to 60 Å as a starting model (Sibanda et al., 2017) (Figure S3). Autorefine in RELION was used to refine the putative single-capped 26S proteasome structure from the manually-picked dataset using the corresponding class reconstruction low-pass filtered to 60 Å as a starting model. The manual picked dataset was used for subsequent analysis using cryoSPARC (Punjani et al., 2017). cryoSPARC was used to *ab initio* reconstruct 5, 10 and 15 3D models. The class corresponding to the 20S proteasome from the 10-model run, containing 3,150 particles, was then subjected to homogeneous refinement using cryoSPARC.

Random particle models were generated using RELION with the template picked particle dataset. Each model was reconstructed using the mean number of particles from the 30 models in Figure 4, ~1000 particles. Particles were sampled without replacement. Model error (E) was calculated for each RELION generated model by taking the harmonic mean of their respective rotational accuracy (R) and translational accuracy (T) as determined using RELION (Equation 2). Model error values were normalized between 1 and 2.

(2) $$E=\frac{2}{\left(\frac{1}{R})+(\frac{1}{T}\right)}$$

We then performed a two-sided Kolmogorov-Smirnov test between the distribution of model error from our models and the distribution of model error from the random particle models.

Several high-abundance complexes from our MS data with identifiable, previously solved structures were used to compare with our top 3 models generated using RELION. All models were first low-pass filtered to 30 Å before being aligned using Chimera’s Fit in Map function (Pettersen et al., 2004). The cross-correlation score was then calculated by using the model with a larger volume as the region of computation, essentially sliding the larger complex across the smaller complex.

Purified proteasomes (a gift from A. Matouschek and C. Davis) were prepared as described above. 80 micrographs were manually recorded and processed using reference-free 2D alignment and classification in RELION.

### QUANTIFICATION AND STATISTICAL ANALYSIS

The statistical tests and associated p values are reported in the figures and/or figure legends for the specific analysis. Distributions of the rotational-translational error for the reconstructed 3D models were compared using a two-sided Kolmogorov-Smirnov test (Figure 4B). For the comparison of the two label-free quantification strategies, each point represents the relative abundance of a given protein complex determined using the two methods (Figure S1B). The Pearson correlation coefficient was then calculated for the resulting data.

### DATA AND SOFTWARE AVAILABILITY

The EM reconstruction for both the 20S and 26S (presented in Figure 5B) were deposited in the EM Data Bank (EMDB) under accession codes EMD-7946, EMD-7947, respectively. The accession number for the MS data reported in this paper is PRIDE: PXD010026.

## Supplementary Material

1

2

## Highlights

- Whole-cell extract can be fractionated and visualized using electron microscopy
- Multiple 3D structures can be recovered from fractionated cell extract
- Mass spectrometry data can inform on the identity of the resulting 3D structures
- Using this method, proteasomes in two different biological states are observed
