# Supplementary material for: Classification of Single Particles from Human Cell Extract Reveals Distinct Structures
Source: Cell Rep. Author manuscript; Available in PMC 2018 Aug 25. (PMC6109231; doi:10.1016/j.celrep.2018.06.022)
Supplement: 1 [file NIHMS981695-supplement-1.pdf]

Cell Reports, Volume *24*

Supplemental Information

Classification of Single Particles from Human Cell Extract Reveals Distinct Structures

Eric J. Verbeke, Anna L. Mallam, Kevin Drew, Edward M. Marcotte, and David W. Taylor

Supplemental Figures:

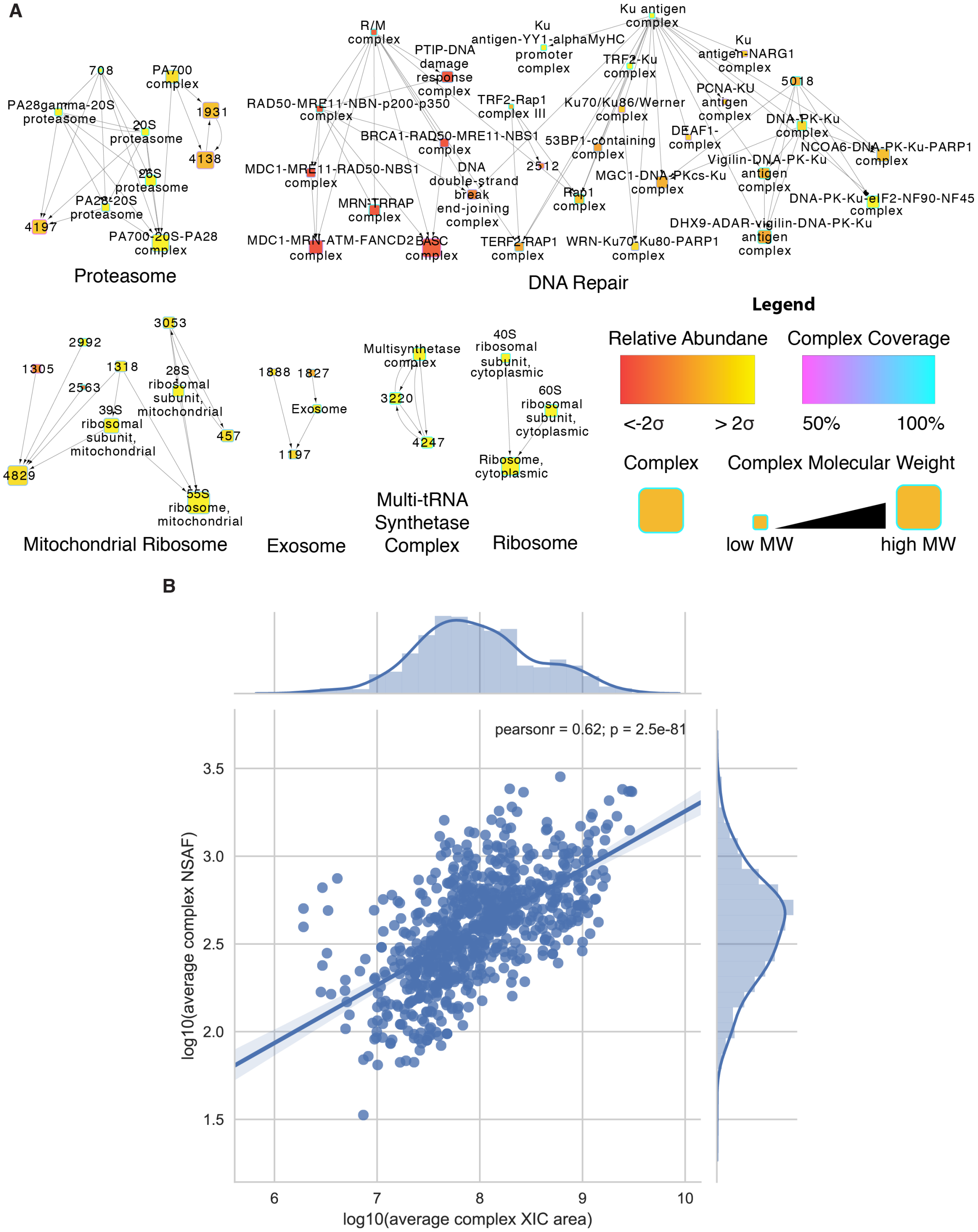

Figure S1. Hierarchical network of related protein complexes. Related to Figure 2 and Table S1.
(A) Subset of the hierarchical network showing related complexes identified by MS in our sample. Each node represents a protein complex and is identified by name or by cluster number from NSAF quantified data (Table S1). The size of each node depicts the molecular weight of the complete complex. Node fill color gradient represents the relative abundance of the complex determined by label-free quantification (see Methods). Node border color gradient represents the percent of subunits in a complex identified by MS. Arrows between nodes indicate at least 90% similarity in subunit composition between source and target node.
(B) Comparison of protein complex relative abundance as calculated using two different label-free quantification strategies.

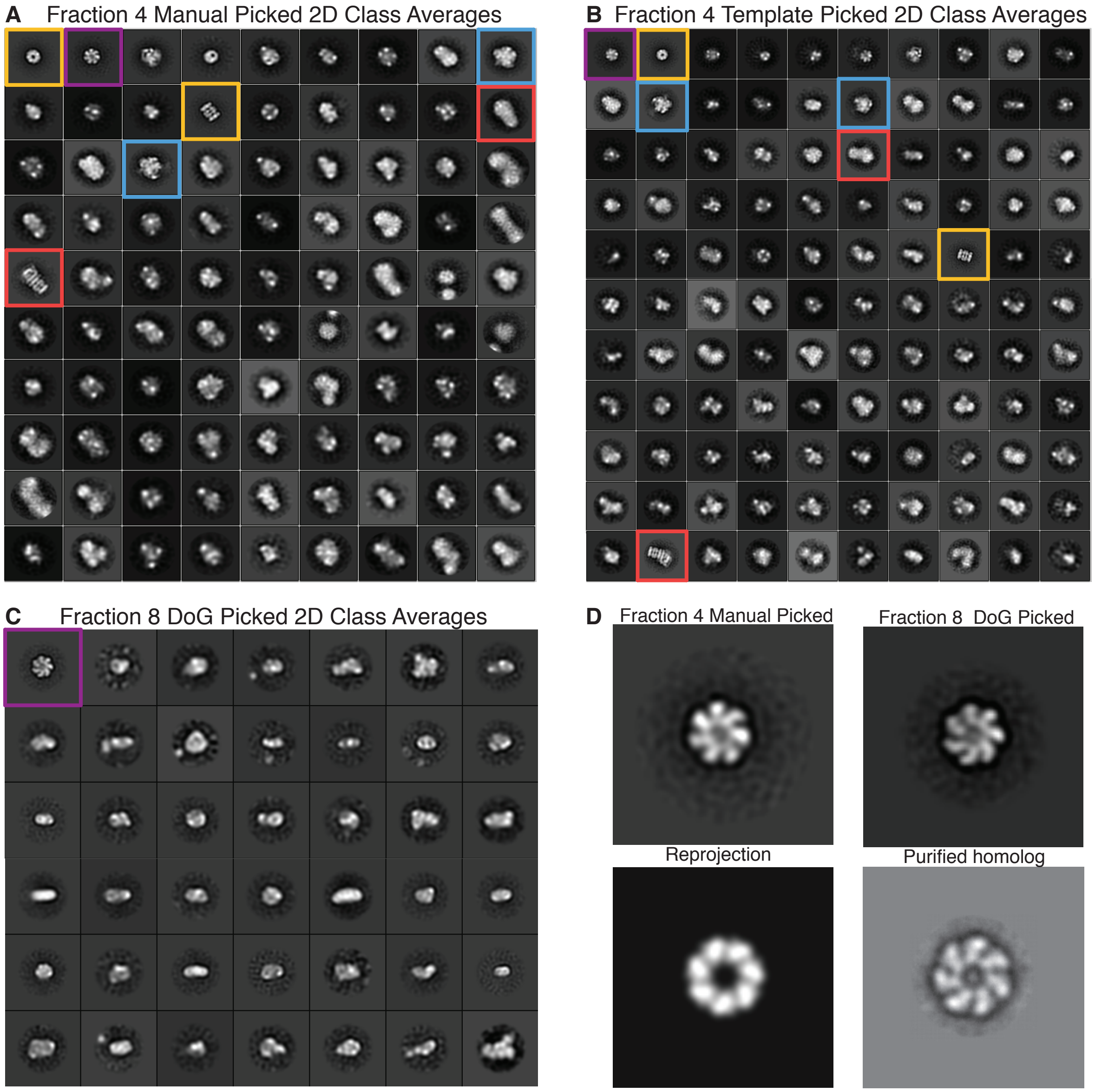

Figure S2. Classification of particles using RELION. Related to Figure 3.
(A) Reference-free 2D class averages of 31,731 template picked particles generated using RELION. The size of each box is 576 Å x 576 Å. The 2D class averages are sorted by the number of particles belonging to each class. Highlighted boxes show examples of similar 2D classes from both particle selection methods of fraction 4 data.
(B) Reference-free 2D class averages of 35,381 manual picked particles generated using RELION. The size of each box is 518.4 Å x 518.4 Å. The 2D class averages are sorted by the number of particles belonging to each class.
(C) Reference-free 2D class averages of 28,553 Difference of Gaussian picked particles generated using RELION. The size of each box is 518.4 Å x 518.4 Å.
(D) Reference-free 2D class averages of HSP60 identified in both fraction 4 and fraction 8. Reprojection of the HSP60 X-ray crystal structure (PDB 4PJ1) low-pass filtered to 30 Å and a 2D class average of a negatively stained purified protein homolog adapted from (Danziger et al., 2003) shown as comparison. Image box sizes are scaled for consistency.

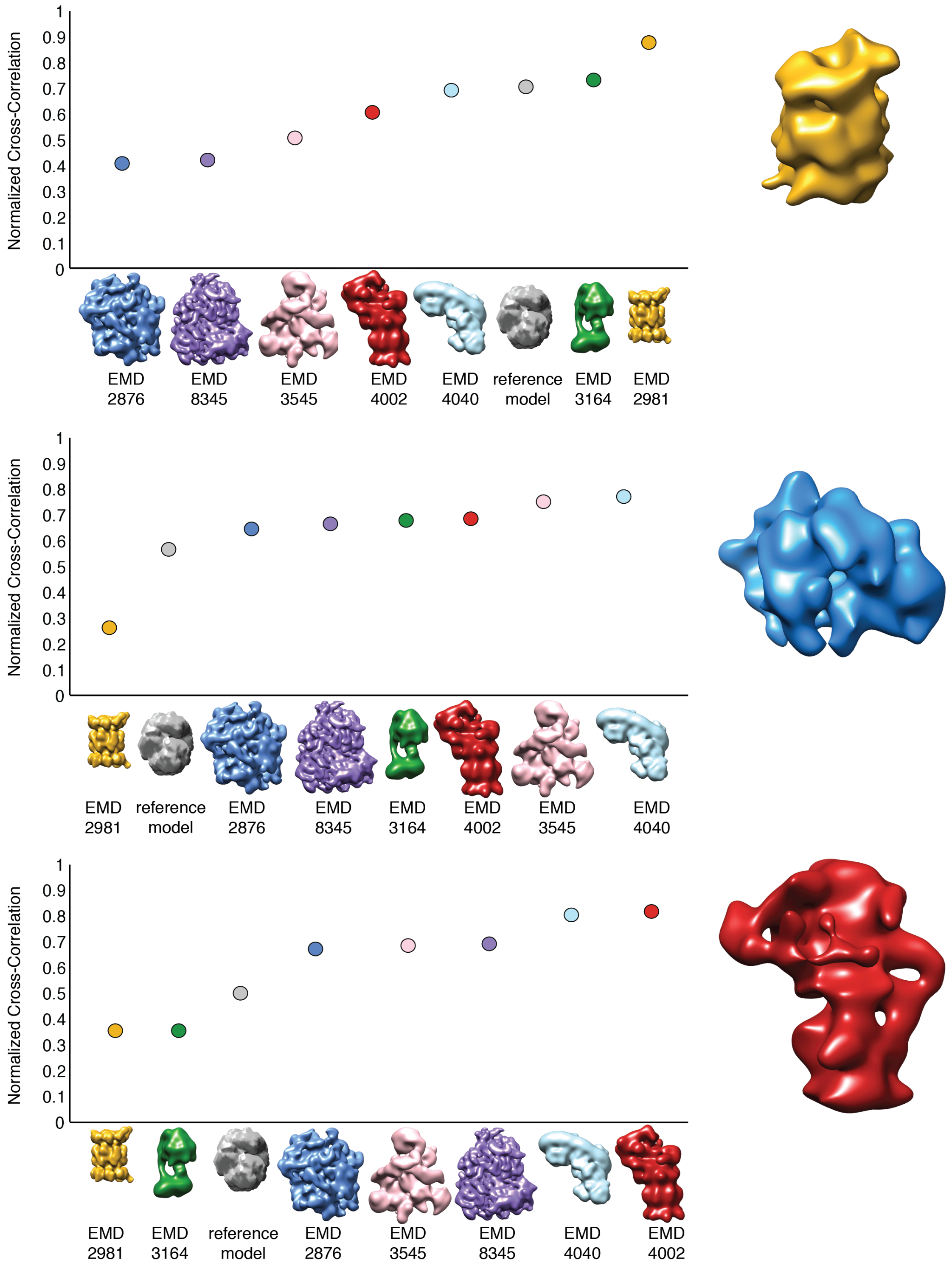

Figure S3. Cross-correlation comparison of top 3 RELION models to complexes identified by MS. Related to Figure 4.

Normalized pairwise cross-correlation scores for our top 3 RELION reconstructions to each of the following previously solved cryo-EM structures: EMD-2876 – mitochondrial ribosome, EMD-2981 – 20S proteasome core,

EMD-3164 – bovine mitochondrial ATP synthase, EMD-3545 – c* spliceosome, EMD-4002 – 26S proteasome, EMD-4040 – respiratory complex I, EMD-8345 – 80S ribosome.

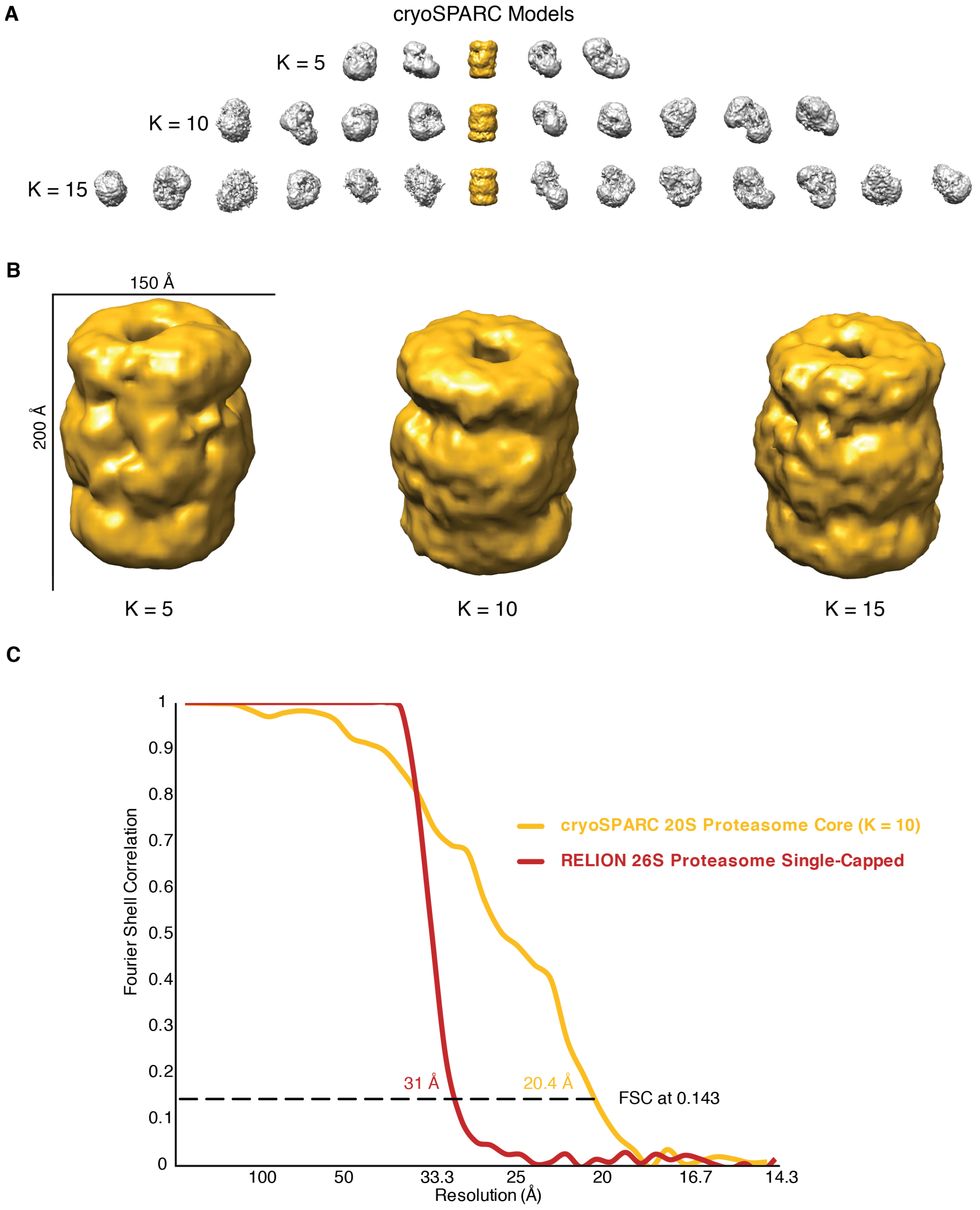

Figure S4. 3D models using cryoSPARC with k = 5,10,15 and related Fourier shell correlations curves. Related to Figure 5.
(A) Reconstructed 3D models from 35,381 manually picked particles when sorted into 5, 10 and 15 *ab initio* classes by cryoSPARC. The 20S proteasome core is highlighted in gold.
(B) Comparison of 20S proteasome core models from 5, 10 and 15 classes.
(C) FSC curves for the single-capped 26S proteasome (red) and 20S core proteasome (gold) shown in Figure 5B. Nominal resolutions were estimated to be 31 Å and 20.4 Å using the 0.143 gold-standard FSC criterion for the single-capped 26S and 20S core proteasome, respectively.

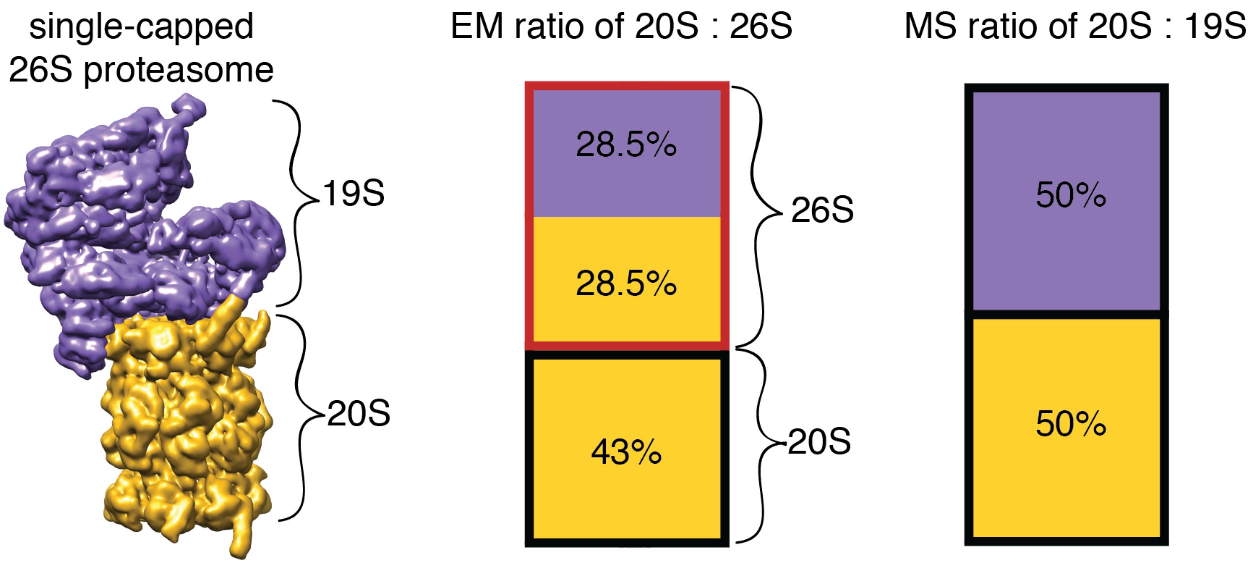

Figure S5. Comparative quantification of the proteasome by MS and EM. Related to Figure 5 and Table S1.
Quantification of proteasome particles by single particle counting of EM data and extracted ion chromatogram areas
